# Efficacy of chemical and biological slug control measures in response to watering and earthworms

**DOI:** 10.1038/s41598-019-39585-5

**Published:** 2019-02-27

**Authors:** Daniel Dörler, Agnes Scheucher, Johann G. Zaller

**Affiliations:** Institute of Zoology, University of Natural Resources and Life Sciences, Vienna, Gregor Mendel-Str. 33, 1180 Vienna, Austria

## Abstract

The Spanish Slug *(Arion vulgaris*, formerly known as *Arion lusitanicus)* is an invasive agricultural and horticultural pest species that causes great damages all over Europe. Numerous options to control this slug are on the market; among the most commonly used are slug pellets containing the active ingredients metaldehyde or iron-III-phosphate and the application of parasitic nematodes (*Phasmarhabditis hermaphrodita*). These control measures potentially also affect non-target organisms like earthworms (Lumbricidae), which themselves can directly and/or indirectly alter a plant’s susceptibility against slug herbivory. Also, the efficacy of slug control treatments is expected to be influenced by watering. In a greenhouse experiment we investigated the influence of daily watering vs. every third day watering on slug control efficacy and potential interactions with earthworms. We found significant interactions between watering and slug control efficacy. Slug herbivory and biomass decreased after application of slug pellets; metaldehyde was more effective under less frequent watering while iron-III-phosphate was unaffected by watering. Parasitic nematodes had no effect on slug herbivory and biomass production. Earthworm activity was reduced with less frequent watering but did not interact with slug control. We conclude that watering patterns should be considered when choosing slug control measures.

## Introduction

Whereas the majority of snail and slug species are not pestiferous, some are causing great damages in agriculture, horticulture and private gardens^1–8^. The invasive slug *Arion vulgaris* Moquin-Tandon 1855 is considered one of the 100 most invasive species in Europe and can nowadays be found almost all over the continent^9,10^ and at various altitudes^11^. Its origin remains unclear^12,13^. Factors that influence slug numbers are frost days in winter and precipitation amount in spring and summer^11,14–16^.

*A*. *vulgaris* has a wide array of feeding plants^1,17–20^. The slug has an annual life cycle^21^ and as hermaphrodites each slug lays around 400 eggs in several clutches from late summer to autumn in potentially frost-sheltered places from where the young slugs hatch after 30–50 days^22^. Most slugs die after oviposition^1,22,23^. After the winter, *Arion* slugs become active whenever air temperatures rise above 5 °C^22^; overall they have shown high adaptability to diverse environmental factors like drought or cold temperatures^24–28^. Especially eggs are very tolerant to substantial water loss, whereas hatched slugs show high activity during moist conditions^29^ but reduce activity during droughts^27^.

To avoid crop damages several slug control measures are available. The most often used chemical substances are applied as pellets containing the active ingredients metaldehyde or iron-III-phosphate^30–38^ and act as food or contact poison^30^. Less often used are biological control measures with the parasitic nematodes *Phasmarhabditis hermaphrodita*^39–45^.

The effectiveness of these slug control measures can be altered by biotic factors such as the size of the slugs^46^ or a slugs’ interaction with other organisms living in the same habitat. Earthworms, for example, have been shown to reduce the efficacy of slug control by consuming slug pellets^47^. Earthworms themselves can also be harmed by consumed molluscicides^48,49^. On the other hand, earthworms can also induce defense mechanisms in plants which makes them less attractive for slugs^50,51^. As slug activity is affected by soil moisture conditions it is interesting to know to what extent slug control measures depend on soil moisture. However, to the best of our knowledge an interaction between these aspects has never been experimentally addressed.

Therefore, the objective of this study was to test the effectiveness of different slug control methods under different irrigation regimes or their interaction with earthworms, as well as potential interactions between irrigation regime and earthworms. We hypothesized that regular irrigation will lead to higher slug activity and lower efficacy of slug control measures. As higher soil moisture will also increase earthworm activity we expected a greater reduction of slug control by earthworms under this condition. These hypotheses were tested in a full-factorial greenhouse experiment using lettuce as model crops.

## Material and Methods

### Experimental Setup

The study was conducted under a 7-x-7-m semi-open rainout shelter located in the town of St. Peter in der Au, Lower Austria (48.044844 N, 14.622733 E) between July 9 and September 22, 2015. We used a three-factorial randomized design using the factors Slug control (4 levels: metaldehyde, iron-III-phosphate, nematodes *P*. *hermaphrodita*, no slug control), Watering regime (2 levels: daily watering with always the same amount of rain water, watering every third day using the same amount as for three single day waterings), and Earthworms (2 levels: absence of earthworms, presence of three adult individuals of the anecic species *Lumbricus terrestris* L.). Each treatment was replicated five times; in total we had 80 experimental units. As experimental units we used 19-l plastic pots (diameter 29 cm, height 29 cm) filled with 14 l of a commercial substrate mixture, consisting of compost soil mixture (Seiringer Umweltservice GmbH, Wieselburg, Austria) and 2.8 l organic peat-free garden substrate (Seramis Bio, Feldbach, Austria). This substrate mixture is often also used by commercial and private gardeners. Characteristics of the soil mixture can be found in Table 1.

**Table 1:** Chemical characteristics and ingredients of used soils.

Parameter	Compost soil mixture of Seiringer (data of analysis)	Seramis Bio Hochbeet-Gemüseerde peat free
pH-value (CaCl_2_)	7.25–8.75	5–7
Salt content (g l^−1^ KCl)	2.6–4.6	1–3
N (mg l^−1^)	45	40–300
P_2_O_5_ (mg l^−1^)	818	40–300
K_2_O (mg l^−1^)	2713	300–1000
Ingredients	green waste compost, fine sand, peat and pieces of red-bricks	bark humus, green waste-compost, horn grit, coconut fiber, calcium carbonate, and extraction residues of oilseeds

To prevent earthworms and slugs from escaping, we taped weed fleece at the bottom of each pot; a 20-cm high plastic-sheet smeared with soft soap at the uppermost 2 cm was taped at the upper rim of each pot.

### Factors Watering Regime and Earthworms

All pots were watered with total 7.2 l of rainwater between July 9–20 to create suitable soil moisture for earthworms and slugs. On July 10, three adult earthworms (*L*. *terrestris*, mean fresh mass of 13.1 ± 1.8 g pot^−1^) were introduced to half of the pots. Earthworms were obtained from a local bait shop (Anglertreff Thomas Lux, Vienna, Austria). The added total earthworm density or biomass translates to 32 individuals m^−2^ or 139 g biomass m^−2^, respectively. Earthworms were fed with 1 g of hay per pot at July 10, 21 and 30; hay was also added to pots without earthworms to keep nutrient addition similar among treatments. In total 9 dead earthworms laying on the soil surface were replaced throughout the experiment.

From July 21 onwards, the different watering patterns were established. Depending on soil moisture 0.1–0.3 l tap water pot^−1^ day^−1^ were given at evening time (July 21–29: 0.3 l pot^−1^, July 30 – August 07: 0.2 l pot^−1^ and August 08 – Sept. 16: 0.1 l pot^−1^). The other half of the pots was watered every three days with the amount of water added to the daily watered pots over three days. This resulted in the same amount of water added to each pot until the end of the experiment.

Ten days after earthworm introduction we planted four lettuce plants (*Lactuca sativa cf*. *Capitata*, *variety Ovation*) in each pot in a consistent pattern.

### Factor Slug Control Treatments

On August 17, 80 sub-adult and 80 adult specimens of *A*. *vulgaris* slugs were hand-collected in a private garden and a meadow near the study site. The collected slugs were kept in plastic jars without food for about 24 hours and introduced to respective pots on August 18. We added one sub-adult (1.9 ± 0.4 g pot^−1^, fresh mass) and one adult slug (6.3 ± 1.3 g pot^−1^). Slugs could freely move in the pots and eat from the lettuce plants.

The following slug control measures were applied on August 18 according to the manuals on the packages:

- META: Metaldehyde (Schneckenkorn Limex ultra, active component 30.0 g kg^−1^ metaldehyde, Scotts Celaflor, Salzburg, Austria): 4 pellets pot^−1^ added on August 18 and September 1.
- FE3P: Iron-III-phosphate (Ferramol Schneckenkorn, active component 9.9 g kg^−1^ iron phosphate; W. Neudorff, Emmerthal, Germany): 15 pellets pot^−1^ added on August 18 and 20. After the application the pots were irrigated as recommended.
- NEMA: Parasitic nematode *P*. *hermaphrodita* (Nemaslug 40 m pack, Save the Plants/Birds, UK). As this product is not registered for use in Austria, we obtained permission from the Federal Agency for Food Safety (permission no. 191.601/01-BAES/2015). As recommended, we applied 0.1 l pot^−1^ of a nematode-water solution on August 18 and 26. Prior to nematodes pots were watered with 0.1 l pot^−1^ tap water as recommended. In total approximately 62.500 infective juvenile nematodes pot^−1^ or 1 million m^−2^ were applied.
- CONT: Control pots did not receive a slug control measure.

The amount of water recommended to accompany slug control measures was also added to all other pots to create similar moisture levels.

### Measurements

Height of lettuce plants (longest leaf from the soil surface) was measured on July 20 and August 10.

On August 14 subsamples of lettuce plants were cut from each pot to calculate the relation between leaf area and biomass and to extrapolate overall lettuce biomass in each pot. Slug herbivory was determined every morning in each pot by estimating the percentage of leaf area eaten by slugs. These percentages were then translated into biomass by using the fresh weight – leaf area ratios calculated earlier. Lettuce was cut at the soil surface and removed from pots on September 9. Afterwards, herbivory was assessed on leaves of fresh lettuce offered day^−1^ pot^−1^ (4.8 ± 2.0 g fresh mass per pot, averaged over 5 days) until September 14. On September 14, all slugs were collected, counted, weighed and conserved in alcohol. The weight of the slugs was calculated by summarizing the weight of all the slugs in each pot at the beginning and the end of the experiment. In a next step we calculated the absolute weight difference for each pot, at which dead slugs were treated like complete loss of weight.

Earthworm activity was measured using the toothpick method (Zaller *et al*. 2014) before slugs were introduced. Therefore, 15 toothpicks pot^−1^ were inserted upright into the surface so earthworms would knock them down when foraging for food. In the morning we counted all toothpicks changed in their original inclination as a measure of earthworm activity. After the introduction of the slugs, we monitored earthworm activity per pot by counting the number of earthworms visible on the soil surface every night, since from that moment on it was not possible to distinguish if toothpicks were knocked down by earthworms or slugs. On September 22 the pots were emptied, and earthworms sorted out, counted and weighed.

Three randomized soil samples were taken using a soil corer (diameter 5 cm, depth 5 cm; ~150 g soil pot^−1^) from all the nematode and control pots to determine nematode abundance. Therefore, we mixed 25 g of each soil sample with 200 ml tap water and extracted the nematodes using an Oostenbrink Elutriator. A random sample of 45 ml was fixed with 5 ml formaldehyde (37.1%) and nematodes counted under a binocular at a magnification of 400x on a 5 ml subsample of this solution.

Soil moisture, soil temperature and soil electrical conductivity were measured daily from July 10 to August 24, and every three days from August 25 to September 14 using a handheld TDR system (TRIME -PICO 64/32, HD2-hand held device; IMKO Micromodultechnik, Ettlingen, Deutschland).

For a more detailed timetable on the activities during the experiment see Table 2.

**Table 2:** Timetable of activities and measurements for the experiment.

Activity	July			August				September		
	Wk 2	Wk 3	Wk 4	Wk 1	Wk 2	Wk 3	Wk 4	Wk 1	Wk 2	Wk 3
Trial setup	✓									
Watering with 2.2 l pot^−1^	✓									
Introduction of 3 adult earthworms pot^−1^	✓									
Watering with 5.2 l pot^−1^ in total	✓	✓								
Addition of 1 g pot^−1^ of hay	✓	✓	✓							
Planting of 4 lettuce plants pot^−1^		✓								
Watering regime applied		✓	✓	✓	✓	✓	✓	✓	✓	
Cutting of lettuce					✓				✓	
Slug introduction & slug control treatment						✓	✓	✓		
TDR measurements	✓	✓	✓	✓	✓	✓	✓	✓	✓	✓
Lettuce growth measurements		✓			✓					
Slug herbivory assessment		✓	✓	✓	✓	✓	✓	✓	✓	
Earthworm activity measurement		✓	✓	✓	✓	✓	✓	✓	✓	✓
Removal of slugs									✓	
Removal of earthworms										✓

Wk = week of respective month in the year 2015. Check marks indicate the time of application or action of the respective activity.

### Statistical analysis

All data were analyzed using R (Version 3.5.1) and R Studio (Version 1.1.456^52^). All data were tested for normality. When normality was confirmed a Levene Test (R package “car”) for equal variances was performed before conducting a three-way ANOVA and Tukey HSD post-hoc tests.

All data deviating significantly from normality were transformed (data on slug herbivory and weight difference of slugs) and afterwards again checked with a Levene Test for equal variances before conducting a three-way ANOVA and Tukey HSD post-hoc tests.

Factors for herbivory, slug weight and nematode activity were slug control treatment, watering regime and earthworm presence; for the analyses of salad biomass factors were earthworm presence and watering regime; for earthworm activity and weight factors were control treatment and watering regime. Soil humidity and soil temperature were included as covariates. Additionally, soil humidity was checked for significant differences between watering regimes using a Mann-Whitney-Wilcoxon-Test.

All graphics were made using the R package “agricolae” and “multcomp”.

### Compliance with ethical standards

We followed all ethical standards based on Austrian law and recommended by the ethics commission of the University of Natural Resources and Life Sciences, Vienna.

## Results

Slug herbivory and slug weight were significantly influenced by slug control but unaffected by the watering regime, earthworms or their interactions (Table 3). The effect of different slug control measures was dependent on watering; there was also a three-way interaction between slug control, watering and earthworms (Table 3).

**Table 3:** Slug herbivory and slug weight in response to slug control (SC), watering regime (W) and earthworms (EW) or their interactions.

Factors	Df	Sum Sq	Mean Sq	F value	Pr(>F)
**Slug herbivory**					
Slug control (SC)	3	18.935	6.312	29.502	5.66E-12***
Watering (W)	1	0.272	0.272	1.272	0.264
Earthworms (EW)	1	0.016	0.016	0.075	0.785
SC:EW	3	1.386	0.462	2.16	0.102
EW:W	1	0.02	0.02	0.095	0.758
SC:W	3	0.598	0.199	0.932	0.431
EW:SC:W	3	0.808	0.269	1.259	0.296
**Slug weight**					
Slug control (SC)	3	380.1	126.68	26.527	3.68E-11***
Watering (W)	1	0.9	0.92	0.194	0.661
Earthworms (EW)	1	2.8	2.81	0.589	0.446
SC:EW	3	14.6	4.86	1.017	0.391
EW:W	1	3.7	3.69	0.774	0.383
SC:W	3	97.7	32.57	6.821	4.79E-4***
EW:SC:W	3	48.2	16.07	3.366	0.024*

Asterisks mark significant effects. Df = degrees of freedom, Sum Sq = sum of squares, Mean Sq = mean square, Pr (>F) = p value.

**Figure 1:**
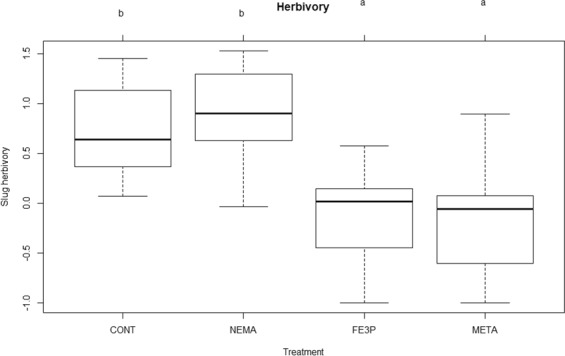
Sum of slug herbivory over all slug control measures. CONT = control group, NEMA = nematode *Phasmarhabditis hermaphrodita*, FE3P = Ferramol (iron-III-phosphate), META = metaldehyde. No influence of earthworms or watering regimes or interaction effects was found. Treatments sharing the same letter are not significantly different.

Iron-III-phosphate and metaldehyde significantly reduced slug herbivory compared to the control group (p < 0.001 for both) and to the nematode treatment (p < 0.001 for both). The nematode group (p = 0.606) did not differ significantly from the control group (Fig. 1).

**Figure 2:**
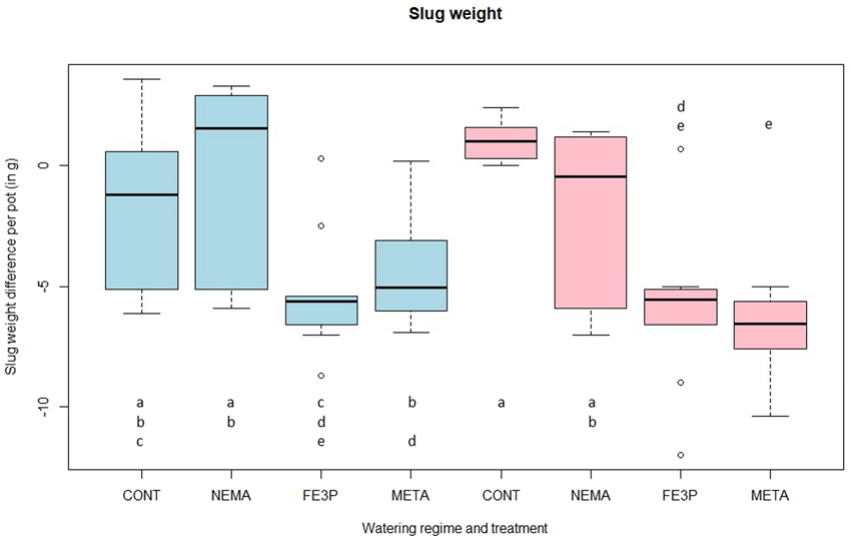
Slug weight pot^−1^ depending on slug control and watering. Blue boxplots mark daily watering, red boxplots mark watering every three days. CONT = control group, NEMA = nematode *P*. *hermaphrodita*, FE3P = Ferramol (iron-III-phosphate), META = metaldehyde. Treatments sharing the same letter are not significantly different.

Iron-III-phosphate (p < 0.001) and metaldehyde (p < 0.001) influenced slug weight significantly compared to the nematode treatment and the control group, which did not differ from each other (both p = 1.000). Additionally, we detected a significant interaction between metaldehyde and watering regime (p = 0.012; Fig. 2). The application of nematodes was neither effective against juvenile (p = 0.668) nor adult *A*. *vulgaris* (p = 0.586).

Mean lettuce biomass at project start was 27.8 ± 12.4 g pot^−1^ and unaffected by slug control, watering regime and earthworm presence or their interactions (Table 4).

**Table 4:** Mean lettuce biomass per pot in response to slug control (SC), watering regime (W) and earthworms (EW) and their respective interactions.

Factors	Df	Sum Sq	Mean Sq	F value	Pr(>F)
Slug control (SC)	3	954	318.1	1.978	0.126
Watering (W)	1	128	127.8	0.795	0.376
Earthworms (EW)	1	23	23.2	0.144	0.705
SC:EW	3	244	81.5	0.507	0.679
SC:W	3	212	70.5	0.439	0.726
EW:W	1	63	63.2	0.393	0.533
SC:W:EW	3	239	79.7	0.495	0.687

Df = degrees of freedom, Sum Sq = sum of squares, Mean Sq = mean square, Pr (>F) = p value.

Number of nematodes in soil samples in pots treated with Nemaslug was significantly higher than in control pots (p < 0.001; in average 50% more nematodes in pots treated with Nemaslug) indicating successful nematode application. Watering regime (p = 0.958) and earthworm presence (p = 0.366) had no significant influence on nematode abundance in soil samples. Soil moisture was not significantly different between the two watering regimes (p = 0.773).

Earthworm weight was neither affected by slug control, nor watering or their interaction (Table 5). Earthworm activity was significantly decreased (p < 0.001) in pots with watering every three days compared to pots with daily watering (Table 5).

**Table 5:** Earthworm weight and activity differences in response slug control and watering and their interaction.

Factors	Df	Sum Sq	Mean Sq	F value	Pr(>F)	
**Earthworm weight**						
Slug control (SC)	3	64.4	21.47	1.363	0.272	
Watering (W)	1	46	46.01	2.921	0.097	
SC:W	3	46.5	15.49	0.984	0.413	
**Earthworm activity**						
Slug control (SC)	3	12.9	4.3	0.153	0.927	
Watering (W)	1	504.1	504.1	17.893	0.000	***
SC:W	3	62	20.7	0.734	0.540	

Df = degrees of freedom, Sum Sq = sum of squares, Mean Sq = mean square, Pr (>F) = p value.

## Discussion

While several studies tested the efficacy of various slug control measures, this is among the first simultaneously investigating their interactions with watering patterns and earthworms. Our results showed a high effectivity of the most widely used slug control methods: pellets containing either metaldehyde or iron-III-phosphate as active ingredients, that is in line with other studies^31–36^. Our results showed that a change in irrigation from daily watering to watering every three days can increase the efficacy of metaldehyde. This is an important information when the aim is to reduce chemical control measures. The reason for this could be that (i) slugs that ate a nonlethal dose of metaldehyde recovered faster in a moister environment than in a drier one, or (ii) a higher amount of watering per event more readily removes active ingredients from metaldehyde pellets. Indeed, it has been shown that heavy rainfalls may also leach metaldehyde into water bodies^53^.

Although the mode of action is different between iron-III-phosphate and metaldehyde and we could not find any effect of watering regime on iron-III-phosphate, similar watering-induced alterations of efficacy could occur and must be further investigated. Clearly, much more ecological research is needed for this control measure which is also allowed for use in organic farming.

The biological control *P*. *hermaphrodita* on the other hand, showed no significant effect on slug herbivory, regardless of the watering regime. This in in contrast to studies showing decreased slug herbivory^34,39–43,45^ or higher slug mortality^39,46^ after nematode applications. As the number of nematodes in pots treated with nematodes was significantly higher than in the control groups, we assumed, that *P*. *hermaphrodita* were active in soil and could possibly infect slugs. Since nematodes prefer moist conditions^41^, we assumed that watering regime would have a significant impact on nematode survival. However, when looking on nematode numbers in the soil we could not find a significant difference between the daily watering and watering every three days. This is in line with another study that showed no effect of *P*. *hermaphrodita* on *A*. *vulgaris*^37^. One explanation for this could be that slugs avoid soil treated with nematodes^54,55^. Since in our experiment slugs could not leave the area and find alternative food sources that are not contaminated with nematodes, they would feed on the lettuce plants. We can also rule out that nematodes did not feed on slugs but on lettuce instead, since they are known to be slug parasites^39^. In contrast to results from another study^46^ we found no effect of *P*. *hermaphrodita* on small specimens of *A*. *vulgaris*. One potential explanation could be that the smallest slugs in our study (1917 mg) were already heavier than the slugs observed to be negatively influenced in other studies^43,44,46^.

We were surprised to find no effect of earthworms on the effectiveness of metaldehyde or iron-III-phosphate as others showed that earthworms removed around 17% of all slug pellets in a field experiment, therefore reducing the effectiveness of the treatment against slugs^47^. An explanation could be that in our experiment food provided in form of hay was more attractive for earthworms than slug pellets or apple leaves as done in other studies^49^. Also, we did not find any negative effects of slug control measures on earthworm activity or earthworm biomass. Such negative effects on both earthworm survival and behaviour after contact with iron-III-phosphate were reported^48,49^. We explain these contrasting results with the fact that Langan and Shaw (2006) exposed earthworms to doses much higher than in the current experiment. Edwards *et al*. (2009) did not only test the slug pellet formulations on the market, but also the pure active ingredients, where they found no negative influences on earthworms. Only together with the chelating agents EDTA (ethylene diamine tetracetic acid) and EDDS (ethylene diamine succinic acid) iron-III-phosphate was found to negatively affect earthworms. So, we suggest that a different chelating agent was used in formulations we tested, however to verify this, further studies seem imperative. We also found no significant effect of earthworms on slug herbivory or weight, which is in contrast to other studies^50,51^, but could be explained by the fact that native plant species (*Arrhenaterum elatius* L.*; Bromus erectus* Huds., *Festuca ovina* L., *Holcus lanatus* L., *Plantago lanceolata* L.*; Knautia arvensis* L., *Leucanthemum ircutianum* Mill., *Prunella vulgaris* L., *Salvia pratensis* L., *Trifolium pratense* L., *Anthyllis vulneraria* L., *Lotus corniculatus* L., *Trifolium pratense* L., *Vicia cracca* L.) were used in the former experiments that could have responded more sensibly to earthworm effects than lettuce in the current experiment. Clearly, more detailed studies would be necessary.

Taken collectively, we found a high efficiency of the chemical control measures metaldehyde or iron-III-phosphate against *A*. *vulgaris*. However, the interaction between slug control and watering on slug weight suggests that more frequent watering could make slug control less effective. The nematode *P*. *hermaphrodita* failed to work against *A*. *vulgaris* in our experiment completely. The finding that slug control did not detrimentally affect earthworm activity and biomass is good news, however demands further studies to elucidate the equivocal findings in the literature. Also, we cannot rule out more subtle effects of slug control measures on smaller soil biota.

## Data Availability

The datasets used and/or analysed during the current study are available from the corresponding author on reasonable request.
